# PARP inhibitor YCH1899 activates type I interferon signaling and synergizes with STING agonists in BRCA-proficient tumors

**DOI:** 10.1186/s43556-026-00553-x

**Published:** 2026-08-26

**Authors:** Li Jia, Ming-Hao Cao, Yu-Ting Sun, Xu-Bin Bao, Wen-Ting Zhu, Yong-Jun Cheng, Li-Min Wang, Hui Yang, Shan-Shan Song, Chang-Qing Tian, Ze-Hong Miao, Chun-Hao Yang, Jin-Xue He

**Affiliations:** 1 State Key Laboratory of Drug Research, Cancer Research Center, Shanghai Institute of Materia Medica, Chinese Academy of Sciences, Shanghai, 201203 China; 2 University of Chinese Academy of Sciences, No.19A Yuquan Road, Beijing, 100049 China; 3 State Key Laboratory of Drug Research, Department of Medicinal Chemistry, Shanghai Institute of Materia Medica, Chinese Academy of Sciences, Shanghai, 201203 China

**Keywords:** PARP inhibitor, YCH1899, STING agonist, Type I interferon, USP20, IRF3 Ser173 phosphorylation

## Abstract

Poly(ADP-ribose) polymerase (PARP) inhibitors are highly effective in BRCA-deficient tumors, yet their therapeutic benefit in BRCA-proficient cancers remains limited. Here, we demonstrate that YCH1899, a next-generation, resistance-overcoming PARP inhibitor, elicits significantly stronger type I interferon signaling than currently approved PARP inhibitors in BRCA-proficient tumor cells. Mechanistically, YCH1899 facilitates the accumulation of cytosolic DNA, thereby robustly activating the cGAS–STING–IRF3 pathway. Consequently, YCH1899 exhibits potent inhibitory effects on the growth of various BRCA-proficient tumors in a STING-dependent manner and enhances the infiltration of CD8⁺ T cells within the tumor microenvironment. To identify tumor-intrinsic regulators of this immune response, we conducted in vivo CRISPR screens, which revealed USP20 as a prominent regulator. The genetic ablation of USP20 markedly diminished the antitumor immunity induced by YCH1899 in vivo, suggesting that USP20 may serve as a potential predictive biomarker. Furthermore, combining YCH1899 with STING agonists synergistically amplifies interferon pathway activation, driven by enhanced phosphorylation of IRF3 at Ser173, resulting in superior tumor control and profound immune cell infiltration and activation within the tumor microenvironment. Collectively, our findings establish YCH1899 as a potent PARP inhibitor capable of extending clinical benefit to BRCA-proficient cancers and provide a rationale for its clinical combination with STING agonists to elicit robust antitumor immunity, even in patients resistant to PARP inhibitors.

**Supplementary Information:**

The online version contains supplementary material available at 10.1186/s43556-026-00553-x.

## Introduction

Poly(ADP-ribose) polymerase (PARP) enzymes catalyze ADP-ribosylation and play central roles in cellular responses to DNA damage, with PARP1 being the predominant family member involved in DNA repair [1]. To date, four PARP inhibitors have been approved by the U.S. Food and Drug Administration (FDA) for the treatment of ovarian, breast, pancreatic, and prostate cancers. In addition to their established use as monotherapies, PARP inhibitors are being extensively investigated in combination regimens, particularly with immune checkpoint inhibitors, to overcome primary and acquired resistance and improve clinical outcomes [2, 3].

Accumulating evidence indicates that PARP inhibitors exert immunomodulatory effects beyond their direct cytotoxicity toward tumor cells [4–7]. By impairing DNA damage repair, PARP inhibition promotes the accumulation of cytosolic DNA fragments, including micronuclei, which are sensed by cyclic GMP-AMP synthase (cGAS) [4, 6, 8, 9]. Activated cGAS produces 2',3'-cyclic GMP-AMP (cGAMP), thereby activating the stimulator of interferon genes (STING) pathway [10, 11]. Downstream STING–IRF3 signaling induces type I interferons and proinflammatory chemokines, such as CXCL10 and CCL5, leading to activation of the type I interferon receptor (IFNAR), JAK–STAT signaling, and upregulation of interferon-stimulated genes (ISGs) [12]. This innate immune response reshapes the tumor microenvironment and promotes T-cell infiltration and activation [6, 13]. Consistent with these observations, PARP inhibitor monotherapy suppresses tumor growth in BRCA-deficient immunocompetent murine models [5, 6]. However, both immune activation and therapeutic efficacy remain limited in BRCA-proficient tumors [7].

Despite these advances, several key questions remain unresolved. The extent to which innate immune activation depends on PARP catalytic inhibition versus PARP1 trapping remains debated, and disentangling their respective contributions has proven challenging [9, 14]. Moreover, although PARP inhibitors synergize with PD-1/PD-L1 blockade in preclinical models [5, 7, 8], phase III trials have generally failed to demonstrate substantial improvements in overall survival [15–19]. These findings underscore the need to better define the determinants of PARP inhibitor-induced immune activation and to identify more effective combination strategies [20].

The cGAS–STING pathway is a central mediator of innate immune sensing in both tumor cells and antigen-presenting cells, making STING an attractive target for cancer immunotherapy. However, the clinical development of STING agonists has been constrained by systemic toxicity and narrow therapeutic windows [21]. Recent studies suggest that combining STING agonists with PARP inhibitors may overcome resistance in BRCA-deficient tumors by enhancing the activity of natural killer cells and tumor-associated macrophages [22, 23], supporting the potential of rational combination strategies.

We previously identified YCH1899 as a next-generation PARP inhibitor capable of overcoming resistance to first-generation PARP inhibitors, with more than 1,000-fold greater catalytic inhibitory potency and approximately 100-fold greater PARP1-trapping activity than olaparib and niraparib [24]. Here, we investigated whether this enhanced trapping activity translates into robust activation of innate immune signaling in BRCA-proficient tumors. Using in vitro and in vivo models, we show that YCH1899 induces DNA damage and activates a STING-dependent type I interferon response, accompanied by increased T-cell infiltration and marked tumor growth suppression. To identify genetic determinants of this immune activation in vivo, we performed pooled loss-of-function CRISPR screening and identified USP20 as a critical regulator of YCH1899-driven interferon signaling and antitumor efficacy. Finally, we demonstrate that YCH1899 potentiates STING agonist-induced interferon responses and antitumor immunity, providing a rationale for combining potent PARP1-trapping activity with STING agonists to extend the therapeutic benefits of PARP inhibition to BRCA-proficient and PARP inhibitor-resistant tumors.

## Results

### YCH1899 primes the innate immune response in BRCA-proficient cells in vitro and in vivo

In BRCA1/2-deficient tumors, PARP inhibition induces DNA damage, activates interferon signaling, and elicits antitumor immune responses [5, 6]. By contrast, how PARP inhibition affects innate immune activation in BRCA-proficient tumors and whether such activation contributes to antitumor efficacy remain insufficiently explored. To address these questions, we examined YCH1899-induced interferon signaling in BRCA-proficient tumor cells and evaluated its immune-dependent antitumor activity in syngeneic mouse models.

**Fig. 1:**
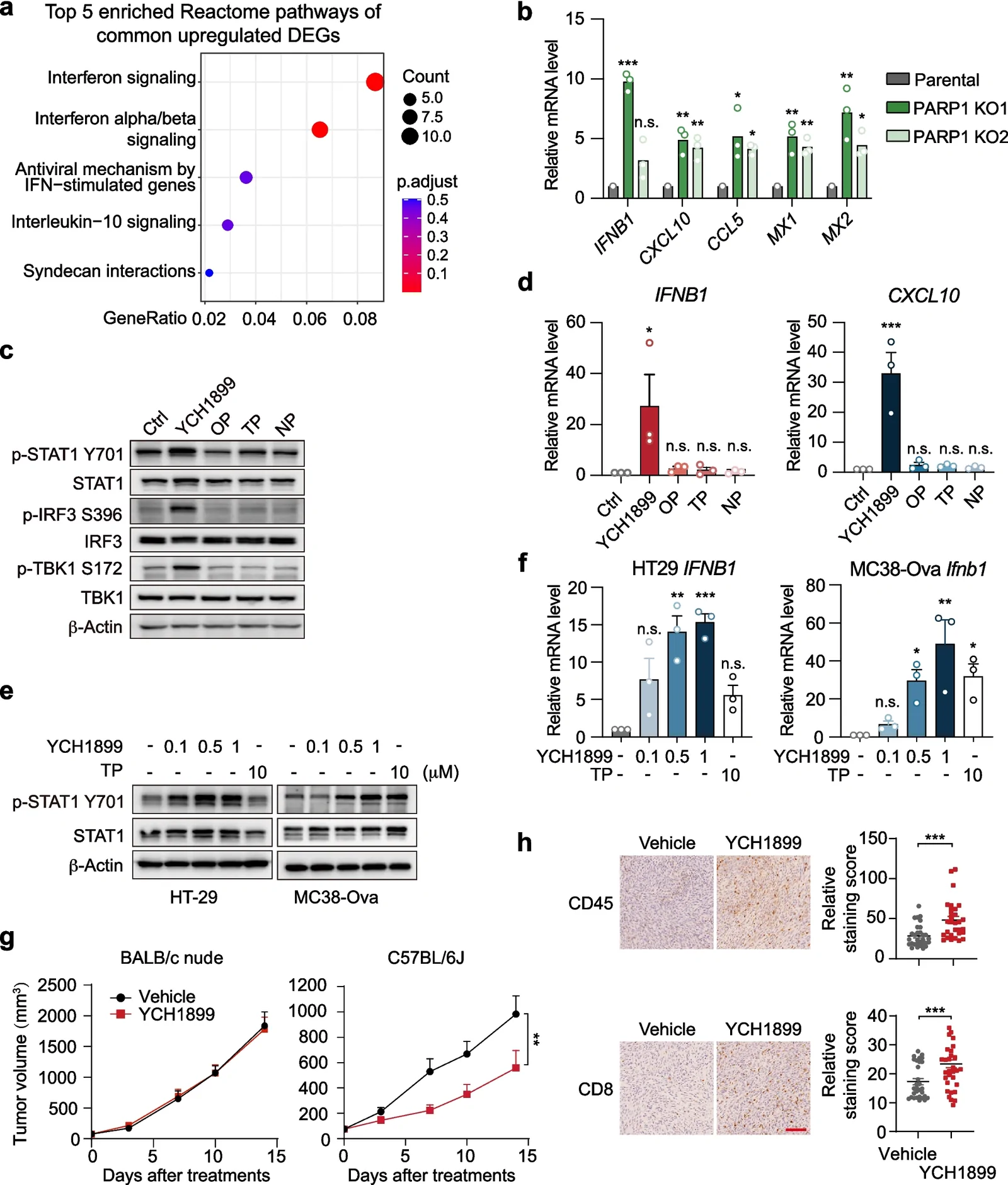
YCH1899 primes the innate immune response in BRCA-proficient cells in vitro and in vivo. **a** Reactome enrichment analysis of commonly upregulated differentially expressed genes (DEGs) in two PARP1-KO HT-29 clones compared with parental cells, as determined by RNA-seq analysis.** b** Upregulation of *IFNB1*, *CXCL10*, *CCL5*, *MX1*, and *MX2* mRNA levels in PARP1-KO HT-29 cells. Data are presented as mean ± SEM. *, *p* < 0.05; **, *p* < 0.01; ***, *p* < 0.001 (n = 3, one-way ANOVA); n.s., not significant.** c** Phosphorylation of STAT1, IRF3, and TBK1 induced by the indicated compounds in HT-29 cells. Cells were treated with 1 μM YCH1899, olaparib (OP), talazoparib (TP), or niraparib (NP) for 48 h.** d** Upregulation of *IFNB1* and *CXCL10* mRNA levels in HT-29 cells treated with 1 μM YCH1899, olaparib (OP), talazoparib (TP), or niraparib (NP) for 48 h. Data are presented as mean ± SEM. *, *p* < 0.05; ***, *p* < 0.001 (n = 3, one-way ANOVA); n.s., not significant.** e** Concentration-dependent increase in STAT1 phosphorylation in HT-29 and MC38-Ova cells treated with the indicated concentrations of YCH1899 or talazoparib (TP) for 48 h.** f** Concentration-dependent upregulation of *IFNB1* (*Ifnb1*) mRNA levels in HT-29 and MC38-Ova cells treated with the indicated concentrations of YCH1899 or talazoparib (TP) for 48 h. Data are presented as mean ± SEM; *, *p* < 0.05; **, *p* < 0.01; ***, *p* < 0.001 (n = 3, one-way ANOVA); n.s., not significant. **g** Growth of MC38-Ova tumors in BALB/c nude and C57BL/6J mice treated orally with vehicle or YCH1899 (15 mg/kg) for 14 days. Data are presented as mean ± SEM; n = 6 mice per group. **, *p* < 0.01 (two-way ANOVA). **h** Immunohistochemical staining for CD45 and CD8 in MC38 tumors treated with vehicle or YCH1899. Scale bar: 100 μm. Quantification of the average staining intensity for CD45 and CD8 is shown. Data are presented as mean ± SEM; n = 10 technical replicates from each of three mice. ***, *p* < 0.001 (unpaired t-test). KO, knockout

We first assessed the effects of genetic PARP1 ablation on innate immune signaling in BRCA-proficient cells. PARP1-knockout HT-29 cells were generated and analyzed by RNA sequencing, quantitative PCR, and immunoblotting. Transcriptomic analysis revealed that PARP1 deletion markedly upregulated ISGs, including *IFI44L*, *MX1*, *MX2*, and *OAS2* (Fig. S1a and Table S1). Pathway enrichment and clustering analyses further demonstrated activation of interferon α/β signaling and interleukin-10-associated pathways following PARP1 loss (Fig. 1a). Consistent with the RNA-seq results, quantitative PCR confirmed increased mRNA levels of *IFNB1*, *CXCL10, CCL5*, and the representative ISGs *MX1* and *MX2* in PARP1-knockout cells (Fig. 1b). Immunoblotting further showed enhanced STAT1 phosphorylation at Tyr701 and Ser727 (Fig. S1b).

We next compared YCH1899 with the FDA-approved PARP inhibitors olaparib, talazoparib, and niraparib for their ability to activate innate immune signaling in BRCA-proficient cells. Among the compounds tested, YCH1899 induced the strongest interferon response, as evidenced by increased phosphorylation of TBK1, IRF3, and STAT1 (Fig. 1c). Correspondingly, YCH1899 significantly upregulated *IFNB1* and *CXCL10* mRNA levels, whereas olaparib, talazoparib, and niraparib had minimal effects at equivalent concentrations (Fig. 1d).

Talazoparib is among the most potent PARP-trapping agents [9]; however, its ability to induce interferon signaling in BRCA-proficient cells appears limited. We therefore performed dose–response analyses comparing YCH1899 with talazoparib in HT-29 and MC38-Ova cells. YCH1899 at 100 nM in HT-29 cells or 500 nM in MC38-Ova cells was sufficient to induce ISG expression and downstream interferon signaling, whereas talazoparib required a substantially higher concentration (10 μM) to achieve comparable effects (Fig. 1e, f; Fig. S1c). Notably, 1 μM YCH1899 produced antiproliferative effects comparable to those of 10 μM talazoparib in both cell lines (Fig. S1d). Together, these data indicate that YCH1899 activates innate immune signaling more efficiently than talazoparib when compared at concentrations producing similar antiproliferative effects.

To determine whether YCH1899-mediated immune activation translates into therapeutic benefit in vivo, MC38-Ova tumors were established in immunocompetent C57BL/6J mice and immunodeficient BALB/c nude mice. YCH1899 significantly suppressed tumor growth in immunocompetent mice, with a treatment-to-control ratio (T/C) value of 58.3% (*p* < 0.01; Fig. 1g). By contrast, YCH1899 showed only limited antitumor activity in nude mice (T/C = 86.3%), indicating that its efficacy depends, at least in part, on an intact immune system (Fig. 1g).

Consistent with these findings, YCH1899 significantly inhibited tumor growth in the MC38 (endpoint T/C = 64.3%) and 4T1 (endpoint T/C = 66.8%) syngeneic models and achieved approximately 20% inhibition of tumor growth in the highly immunosuppressive LL/2 model (Fig. S1e). Immunohistochemical analysis revealed increased infiltration of CD45⁺ leukocytes and CD8⁺ T cells following YCH1899 treatment (Fig. 1h). Collectively, these data demonstrate that YCH1899 activates interferon signaling and elicits immune-dependent antitumor responses in vitro and in vivo*,* highlighting its therapeutic potential in BRCA-proficient tumors.

### YCH1899 activates the STING pathway in BRCA-proficient tumors

Given the activation of interferon signaling and increased T-cell infiltration following YCH1899 treatment, we next investigated the molecular mechanisms underlying this immune response.

**Fig. 2:**
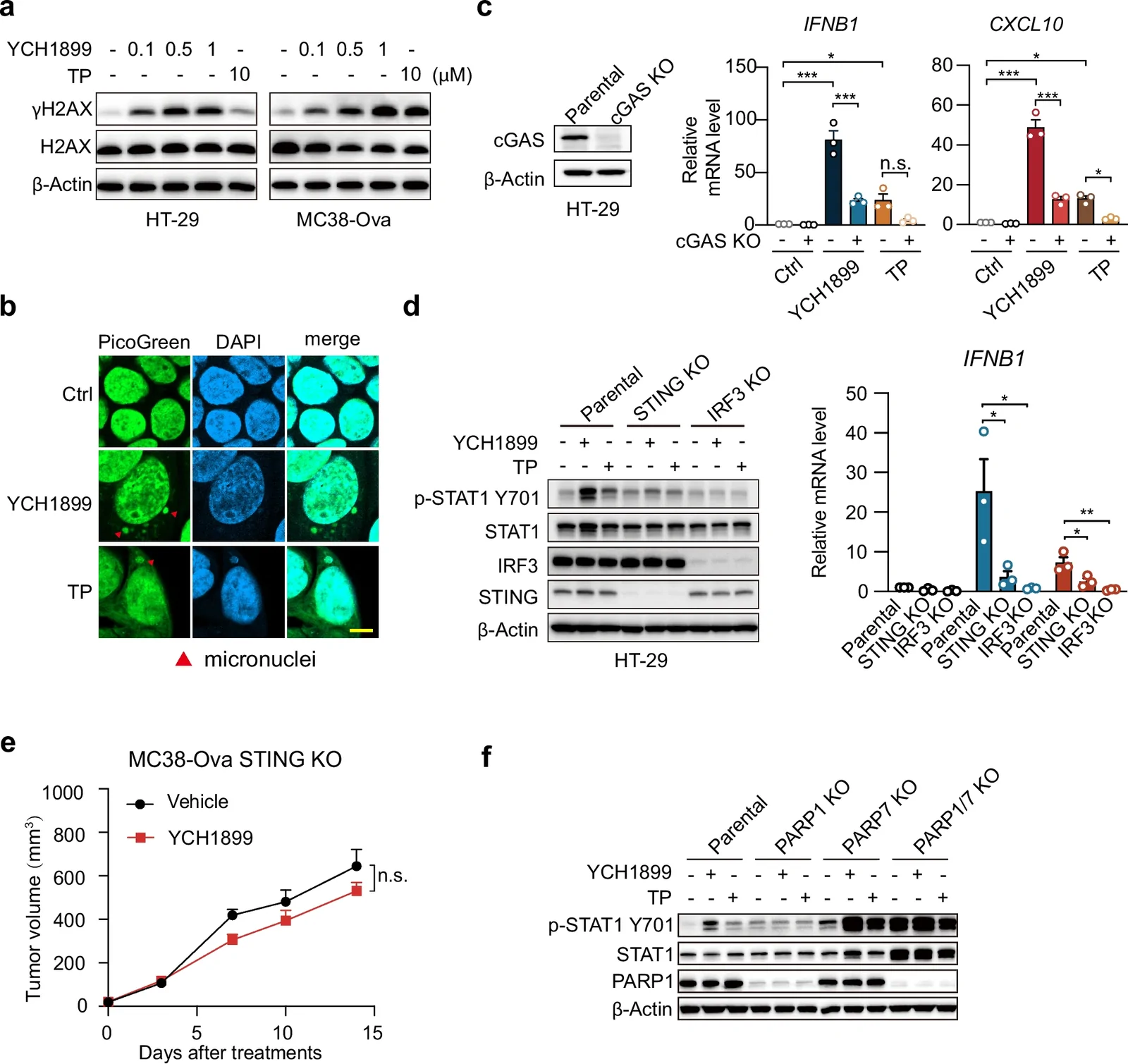
YCH1899 induces DNA damage and activates the STING pathway in BRCA-proficient tumors. **a** Accumulation of DNA damage in HT-29 and MC38-Ova cells following treatment with the indicated concentrations of YCH1899 or talazoparib (TP) for 48 h.** b** Microscopic analysis of cytosolic micronuclei in HT-29 cells treated with vehicle, 1 μM YCH1899, or 10 μM talazoparib (TP) for 48 h. Representative images from three independent experiments are shown, with PicoGreen (green; micronuclei) and DAPI (blue; nuclei) staining. Red triangles denote micronuclei. Scale bar: 10 μm.** c** cGAS-KO abolished YCH1899-induced upregulation of *IFNB1* and *CXCL10* mRNA levels. Left, Western blot confirmation of cGAS-KO; right, cells were treated with 1 μM YCH1899 or 10 μM talazoparib (TP) for 48 h. Data are presented as mean ± SEM. *, *p* < 0.05; ***, *p* < 0.001 (n = 3, one-way ANOVA); n.s., not significant.** d** STING-KO or IRF3-KO impaired YCH1899-induced activation of the interferon pathway. Cells were treated with 1 μM YCH1899 or 10 μM talazoparib (TP) for 48 h. Right, data are presented as mean ± SEM. *, *p* < 0.05; **, *p* < 0.01 (n = 3, one-way ANOVA).** e **Growth of STING-KO MC38-Ova tumors in C57BL/6J mice treated orally with vehicle or YCH1899 (15 mg/kg) for 13 days. Data are presented as mean ± SEM; n = 6 mice per group; n.s*.,* not significant (two-way ANOVA). **f** PARP1, but not PARP7, was essential for YCH1899-mediated activation of the interferon pathway. Cells were treated with 1 μM YCH1899 or 10 μM talazoparib (TP) for 48 h. KO, knockout

YCH1899 robustly activated the STING signaling cascade, as evidenced by increased phosphorylation of TBK1 and IRF3 (Fig. 1c). We therefore examined whether this activation was associated with DNA damage and cytosolic DNA accumulation. The results showed that YCH1899 induced DNA damage in a dose-dependent manner, as indicated by increased γ-H2AX levels in BRCA-proficient HT-29 and MC38-Ova cells (Fig. 2a). Talazoparib similarly increased γ-H2AX levels at 10 μM. Consistent with the induction of genomic instability, YCH1899 also promoted micronucleus formation, as detected by PicoGreen staining in HT-29 cells (Fig. 2b).

To determine whether YCH1899-induced interferon signaling depends on cytosolic DNA sensing, we generated cGAS-knockout HT-29 cells. cGAS deletion markedly attenuated the YCH1899-induced upregulation of *IFNB1* and *CXCL10* mRNA levels (Fig. 2c). Furthermore, genetic ablation of *STING* or *IRF3* substantially impaired the induction of *IFNB1* and *CXCL10* and abolished STAT1 phosphorylation following YCH1899 treatment (Fig. 2d and Fig. S2a, b and c). Together, these findings demonstrate that YCH1899-induced interferon signaling requires an intact cGAS–STING–IRF3 pathway.

To assess the contribution of tumor-intrinsic STING signaling to antitumor activity in vivo, STING-knockout MC38-Ova tumor cells were implanted into C57BL/6J mice, which were subsequently treated with YCH1899. The antitumor efficacy of YCH1899 was markedly reduced in STING-deficient tumors (Fig. 2e), demonstrating that the cGAS–STING–TBK1–IRF3 pathway contributes to YCH1899-induced type I interferon signaling and antitumor activity in BRCA-proficient tumor models.

YCH1899 is a highly potent PARP1 inhibitor, with an IC₅₀ below 0.001 nM and more than 7,000-fold selectivity for PARP1 over PARP7 (IC₅₀ = 7.3 nM) [24]. Because both PARP1 and PARP7 have been implicated in the regulation of type I interferon responses [25, 26], we sought to identify the PARP isoform responsible for YCH1899-mediated immune activation. Using CRISPR/Cas9-mediated gene editing, we selectively ablated PARP1 or PARP7 in HT-29 cells [27]. Loss of PARP1, but not PARP7, significantly reduced STAT1 phosphorylation and *IFNB1* mRNA induction in response to YCH1899 or talazoparib (Fig. 2f and Fig. S2d). By contrast, the PARP7 inhibitor RBN-2397 induced *IFNB1* expression in parental and PARP1-deficient cells but not in PARP7-deficient cells (Fig. S2d).

Overall, these results establish that YCH1899 promotes cytosolic DNA accumulation and activates a STING-dependent type I interferon response in BRCA-proficient cells through a predominantly PARP1-dependent mechanism.

### In vivo CRISPR screening identifies USP20 as a critical regulator of YCH1899-induced antitumor immunity

**Fig. 3:**
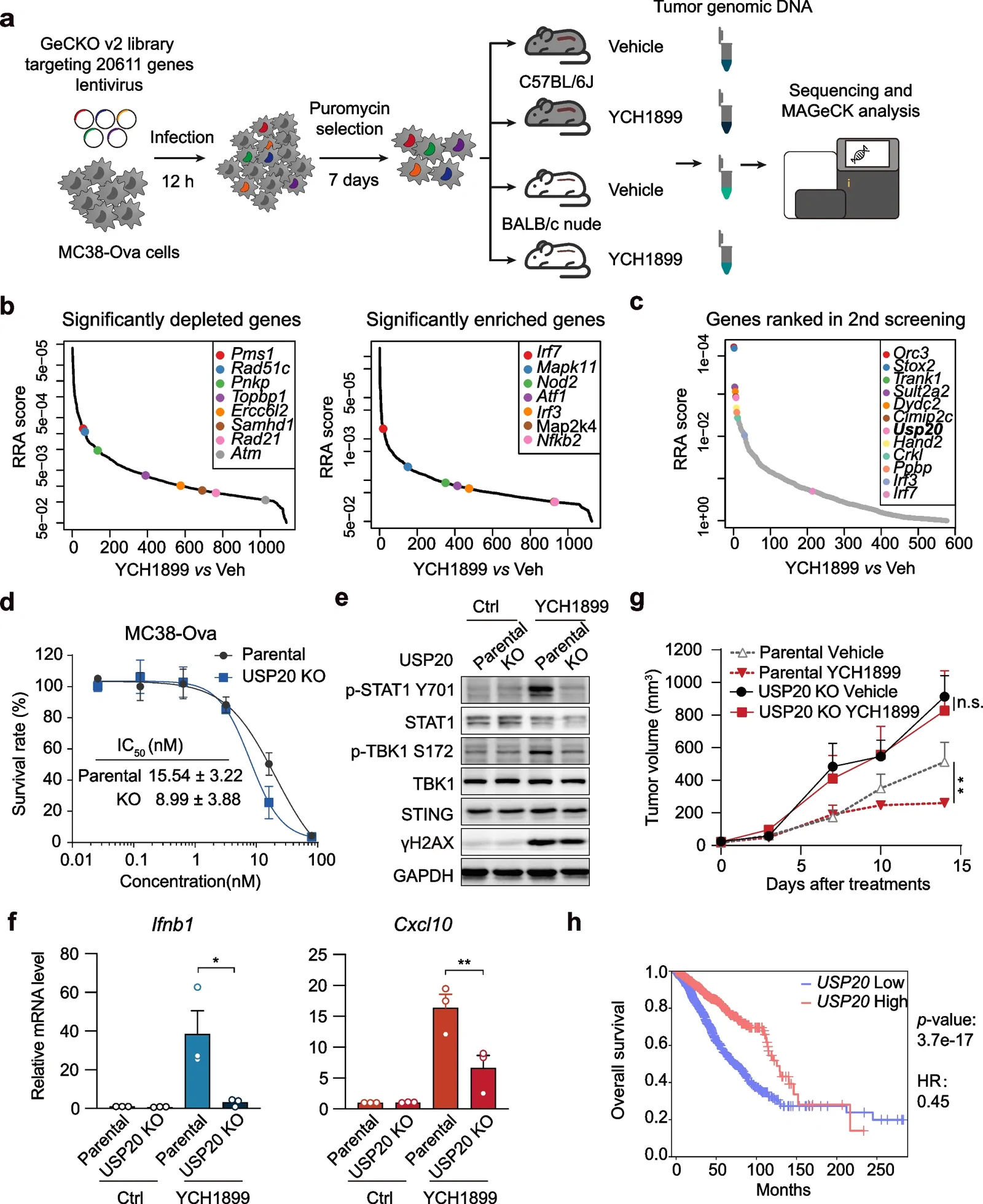
In vivo CRISPR screening identifies USP20 as a critical regulator of YCH1899-induced antitumor immunity. **a** Workflow of the in vivo CRISPR screen. **b** RRA score ranking of genes significantly depleted or enriched (*p* < 0.05) in the genome-wide screen in C57BL/6J mice, as analyzed by MAGeCK. Ranked dot plots illustrate the depleted (left) and enriched (right) genes in YCH1899-treated groups compared with vehicle-treated controls. **c** RRA score ranking of significantly (*p* < 0.05) enriched genes in the secondary screen, analyzed using MAGeCK. Ranked dot plots display enriched genes in YCH1899-treated groups versus vehicle-treated controls. **d** USP20 deletion did not affect the cytotoxic effects of YCH1899. Parental and USP20-KO MC38-Ova cells were exposed to YCH1899 for 6 days. Data are presented as mean ± SEM, and IC_50_ values are shown as mean ± SD (n = 3). **e** USP20 deletion suppressed YCH1899-induced activation of the interferon pathway. Parental and USP20-KO MC38-Ova cells were treated with 1 μM YCH1899 for 48 h. **f** USP20 deletion suppressed YCH1899-induced upregulation of *Ifnb1* and *Cxcl10* mRNA levels. Parental and USP20-KO MC38-Ova cells were treated with 1 μM YCH1899 for 48 h. Data are presented as mean ± SEM; *, *p* < 0.05; **, *p* < 0.01 (n = 3, Student’s t-test). **g** Growth of parental and USP20-KO MC38-Ova tumors in C57BL/6J mice treated orally with vehicle or YCH1899 (15 mg/kg) for 14 days. Data are presented as mean ± SEM; n = 6 mice per group; **, *p* < 0.01 (two-way ANOVA); n.s., not significant. **h** Overall survival analysis according to *USP20 *expression in breast, pancreatic, prostate, and ovarian cancers using GEPIA 3. KO, knockout

To identify tumor-intrinsic determinants of the antitumor immune response elicited by YCH1899, we performed an in vivo CRISPR–Cas9 loss-of-function screen (Fig. 3a). MC38-Ova cells were transduced with the genome-wide mouse GeCKO v2 sgRNA library, which contains 67,405 sgRNAs targeting 20,611 genes [28]. Following puromycin selection and in vitro expansion to allow efficient gene editing, the library-transduced cells were implanted into immunocompetent C57BL/6J mice or, in parallel, into immunodeficient BALB/c nude mice lacking functional CD4⁺ and CD8⁺ T cells. Tumor-bearing mice were treated with vehicle or YCH1899 (15 mg/kg), and tumors were harvested after 14 days for sgRNA representation analysis.

Comparative analysis using MAGeCK identified sgRNAs targeting 1,127 significantly enriched genes and 1,139 significantly depleted genes (*p* < 0.05) in tumors from YCH1899-treated immunocompetent mice relative to vehicle-treated controls (Fig. 3b and Table S2) [29, 30]. Notably, sgRNAs targeting multiple components of the interferon signaling pathway, including *Irf3*, *Irf7*, *Nod2*, and *Map2k4*, were significantly enriched in tumors from YCH1899-treated immunocompetent mice but not in those from nude mice (Fig. 3b and Fig. S3a). These findings highlight a central role for interferon signaling in YCH1899-induced antitumor immunity and are consistent with our observation that IRF3 deficiency abolishes the interferon response to YCH1899 (Fig. 2d). Conversely, sgRNAs targeting genes involved in DNA damage repair, including *Rad51c*, *Pnkp*, and *Atm*, were significantly depleted following YCH1899 treatment (Fig. 3b), suggesting that loss of these genes sensitizes tumor cells to PARP inhibition, consistent with previous reports [31–33].

To validate and refine these findings, we performed a secondary in vivo screen using a customized sgRNA library targeting 578 genes enriched in the primary screen (*p* < 0.05; ≥ 2 positive sgRNAs per gene; essential genes and miRNAs excluded). This focused screen identified 41 significantly enriched genes (*p* < 0.05), including *Irf3* (Fig. 3c and Table S3). Among these candidates, *Usp20* emerged as a prominent hit (Fig. 3c). Given the reported roles of USP20 in antiviral immunity and cancer [34, 35], we next investigated its functional relevance to YCH1899-mediated immune activation.

USP20-knockout MC38-Ova cells were generated using CRISPR-Cas9 (Fig. S3b). USP20 loss did not alter the in vitro cytotoxicity of YCH1899, as indicated by comparable IC₅₀ values in parental and knockout cells (Fig. 3d). By contrast, USP20 deficiency markedly attenuated YCH1899-induced *Ifnb1* and *Cxcl10* expression and reduced STAT1 phosphorylation (Fig. 3e, f), indicating impaired interferon signaling. Consistent with these findings, YCH1899 significantly inhibited the growth of parental MC38-Ova tumors but showed substantially reduced antitumor activity against USP20-deficient tumors (Fig. 3g).

Because ubiquitin-specific peptidases regulate protein stability, we performed quantitative proteomic analysis of parental and USP20-knockout MC38-Ova cells to identify candidate downstream proteins involved in immune regulation (Table S4). Multiple proteins associated with antigen presentation, including DPY30, PJA1, and SERPINE1, were significantly downregulated in USP20-deficient cells (Fig. S3c). The underlying mechanism by which these proteins contribute to USP20-mediated immune regulation remains to be further elucidated. Together, these results identify USP20 as a critical mediator of the interferon-dependent antitumor immune response induced by YCH1899 in vivo. More importantly, survival analysis across ovarian, breast, pancreatic, and prostate cancers revealed that low *USP20* expression is associated with poor survival (Fig. 3h) [36]. This association supports the potential prognostic relevance of USP20 and warrants its further evaluation as a predictive biomarker for PARP inhibitor response.

### Combining YCH1899 with STING agonist treatment enhances interferon signaling

**Fig. 4:**
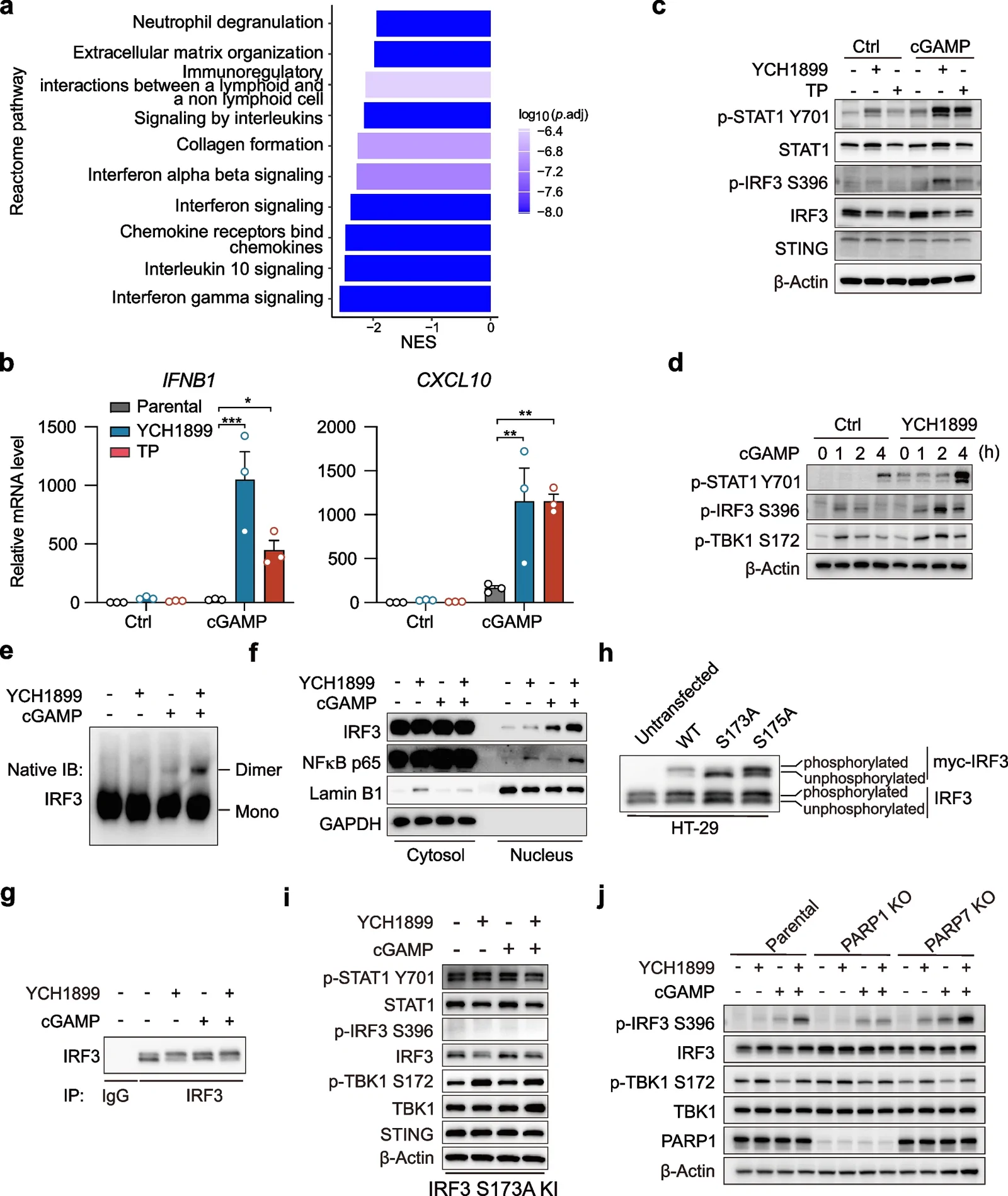
Combining YCH1899 with STING agonist treatment enhances interferon signaling. **a** Reactome pathway analysis of resistance signatures from CTR-DB (CTR-DB ID: CTR_Microarray_161-I). NES, normalized enrichment score. **b** YCH1899 and talazoparib upregulated *IFNB1* and *CXCL10* mRNA levels in response to cGAMP. HT-29 cells were treated with 1 μM YCH1899 or 10 μM talazoparib (TP) for 48 h, followed by cGAMP stimulation for 4 h. Data are presented as mean ± SEM; *, *p* < 0.05; **, *p* < 0.01; ***, *p* < 0.001 (n = 3, one-way ANOVA). **c** YCH1899 and talazoparib enhanced cGAMP-induced activation of the interferon signaling pathway. HT-29 cells were treated with 1 μM YCH1899 or 10 μM talazoparib (TP) for 48 h, followed by cGAMP stimulation for 4 h. **d** Activation of the interferon signaling pathway was assessed at the indicated time points after cGAMP stimulation. HT-29 cells were pretreated with 1 μM YCH1899 for 48 h before cGAMP stimulation. **e** IRF3 dimerization in HT-29 cells following combined treatment with YCH1899 and cGAMP. Cells were treated with 1 μM YCH1899 for 48 h, followed by cGAMP stimulation for 2 h. **f** Nuclear translocation of IRF3 in HT-29 cells treated with YCH1899 and cGAMP. Cells were treated with 1 μM YCH1899 for 48 h, followed by cGAMP stimulation for 2 h. **g** Immunoprecipitation of IRF3 in HT-29 cells treated with 1 μM YCH1899 for 48 h, followed by cGAMP stimulation for 2 h. **h** The IRF3 S173A mutation diminished the upper phosphorylated IRF3 band. HT-29 cells were transfected with myc-IRF3, myc-IRF3 S173A, or myc-IRF3 S175A vectors for 48 h. **i** The combinatorial effects were dampened in HT-29 IRF3 S173A knock-in (KI) cells. Cells were treated with 1 μM YCH1899 for 48 h, followed by cGAMP stimulation for 4 h. **j** PARP1, but not PARP7, was required for the activation of the interferon signaling pathway in response to combined treatment with YCH1899 and cGAMP. Parental, PARP1-KO, and PARP7-KO HT-29 cells were treated with 1 μM YCH1899 for 48 h, followed by cGAMP stimulation for 2 h. KO, knockout

Although YCH1899 activated interferon signaling in BRCA-proficient tumor cells, its tumor-inhibitory effect could be further enhanced. Moreover, an immunosuppressive tumor microenvironment can limit the efficacy of PARP inhibitors even in BRCA-deficient tumors. Consistent with this notion, analysis of clinical samples from veliparib-treated patients revealed reduced interferon pathway activation in the resistant group (Fig. 4a). We therefore hypothesized that STING agonists could potentiate the antitumor effects of YCH1899 and evaluated the combined effects of YCH1899 and cGAMP, an endogenous STING agonist, on interferon signaling.

In HT-29 cells, pretreatment with YCH1899 markedly potentiated cGAMP-induced *IFNB1* and *CXCL10* mRNA expression compared with cGAMP alone (Fig. 4b). YCH1899 pretreatment also enhanced cGAMP-induced phosphorylation of STAT1 and IRF3, whereas talazoparib produced a similar but less pronounced effect (Fig. 4c). Time-course analysis revealed rapid activation of the STING cascade following combination treatment, with TBK1 and IRF3 phosphorylation detectable as early as 1 h, followed by STAT1 phosphorylation at later time points (Fig. 4d).

To further characterize IRF3 activation, we examined its dimerization and subcellular localization. Native PAGE analysis demonstrated increased IRF3 dimerization following combined treatment with YCH1899 and cGAMP (Fig. 4e). Consistently, nuclear/cytoplasmic fractionation revealed enhanced nuclear translocation of IRF3 in cells receiving the combination treatment compared with either agent alone (Fig. 4f). These results indicate that YCH1899 amplifies cGAMP-induced STING signaling by promoting IRF3 activation.

Interestingly, IRF3 appeared as two distinct bands under basal conditions. YCH1899 selectively increased the abundance of the upper, slower-migrating IRF3 species, whereas cGAMP stimulation alone had a much weaker effect (Fig. 4c). Immunoprecipitation analysis further supported increased phosphorylation of this upper IRF3 species following YCH1899 treatment (Fig. 4g). Notably, the enhancement of the upper IRF3 band did not correspond to canonical phosphorylation at Ser396, suggesting the involvement of noncanonical phosphorylation sites. To identify potential residues, mass spectrometric analysis of the immunoprecipitated upper IRF3 band identified a phosphorylation site at either Ser173 or Ser175 (Fig. S4a). To evaluate these sites, IRF3 mutants carrying serine-to-alanine substitutions at Ser173 or Ser175 were expressed in HT-29 cells. The S173A mutation markedly reduced the upper IRF3 band, whereas the S175A mutation had no appreciable effect (Fig. 4h). A similar pattern was observed in 293T and HeLa cells (Fig. S4b), identifying Ser173 as the primary determinant of the upper IRF3 species.

We next examined whether IRF3 Ser173 was required for the enhanced interferon response to combination treatment. An IRF3 S173A knock-in HT-29 cell line was generated in which one allele was disrupted and the other carried the S173A substitution (Fig. S4c). In these cells, STAT1 phosphorylation induced by either YCH1899 or cGAMP alone was reduced (Fig. 4i). Importantly, the enhancement of STAT1 phosphorylation by combination treatment was also markedly attenuated, and IRF3 Ser396 phosphorylation was barely detectable. These findings indicate that Ser173 is critical for the enhanced interferon response. Together, the data support a model in which YCH1899 promotes IRF3 phosphorylation at Ser173, priming IRF3 for a stronger response to cGAMP-induced STING signaling, thereby amplifying interferon pathway activation.

Importantly, genetic ablation of PARP1, but not PARP7, abolished the enhanced IRF3 activation observed following combination treatment (Fig. 4j), demonstrating that PARP1 is required for the potentiation of the STING–IRF3 axis by YCH1899.

Together, these findings establish that YCH1899 primes STING signaling through a PARP1-dependent mechanism and thereby sensitizes tumor cells to STING agonist-mediated immune activation.

### YCH1899–STING agonist combination therapy enhances antitumor efficacy in vivo

**Fig. 5:**
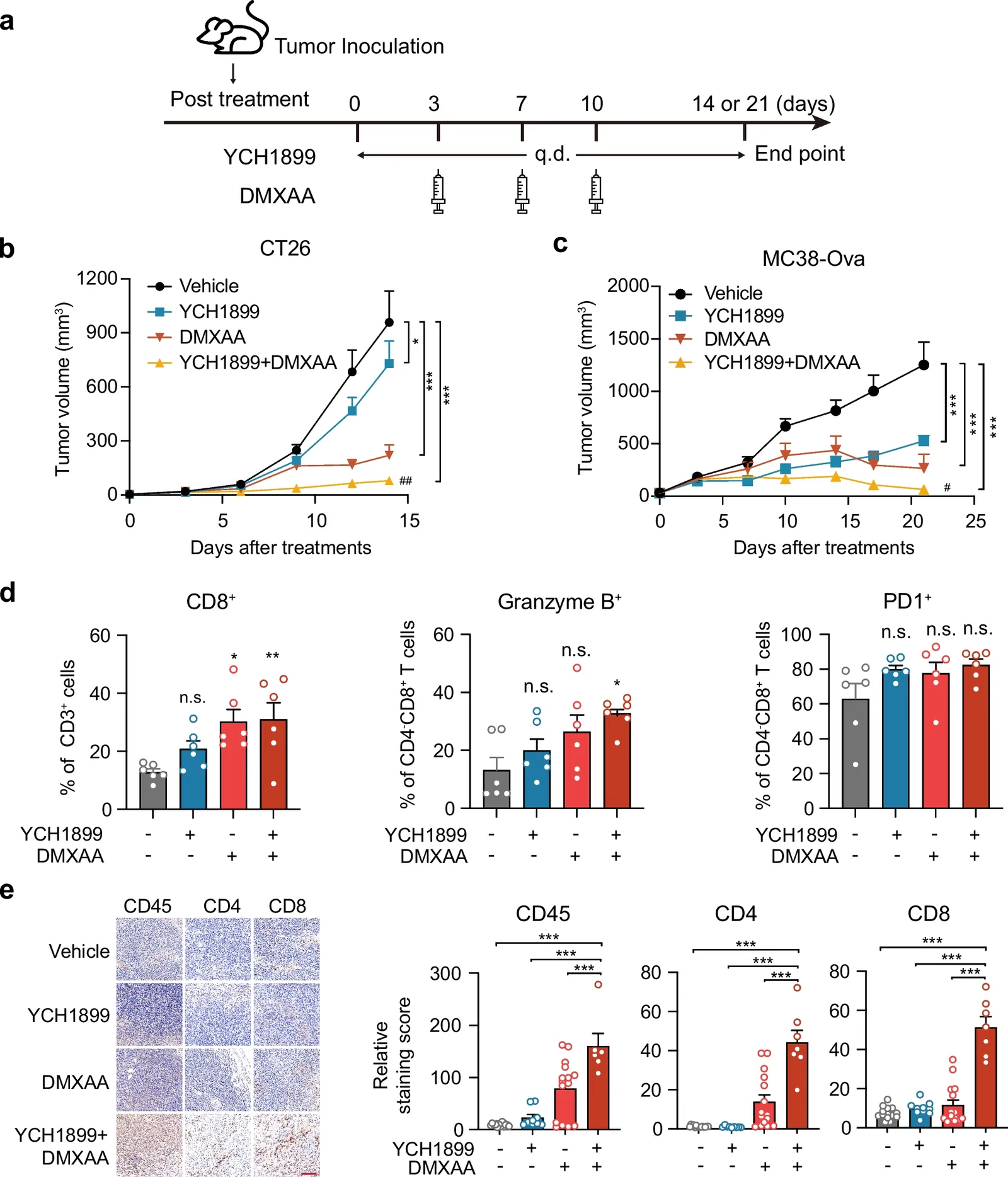
YCH1899–DMXAA combination therapy enhances antitumor efficacy and immune responses in vivo.** a** Schematic of the administration schedule for combined treatment with YCH1899 and DMXAA.** b** Growth of CT26 tumors in BALB/c mice treated with oral YCH1899 (15 mg/kg) for 14 days, intratumoral DMXAA (250 μg) on days 3, 7, and 10, or the combination. Data are presented as mean ± SEM; n = 6 mice per group; *, *p* < 0.05; ***, *p* < 0.001 (two-way ANOVA); n.s., not significant. Combination efficacy at the endpoint was analyzed using invivoSyn; ^##^, *p* < 0.01 (HSA model). **c** Growth of MC38-Ova tumors in C57BL/6J mice treated with oral YCH1899 (15 mg/kg) for 21 days, intratumoral DMXAA (250 μg) on days 3, 7, and 10, or the combination. Data are presented as mean ± SEM; n = 6 mice per group; ***, *p* < 0.001 (two-way ANOVA). Combination efficacy at the endpoint was analyzed using invivoSyn; ^#^, *p* < 0.05 (HSA model). **d** Combination treatment with YCH1899 and DMXAA promoted CD8^+^ T-cell infiltration and activation. Tumor tissues were collected from the CT26 model at the study endpoint. Data are presented as mean ± SEM; n = 6 mice per group; *,* p* < 0.05; **, *p* < 0.01 (one-way ANOVA); n.s., not significant.** e** Immunohistochemical analysis of CD45, CD4, and CD8 in MC38-Ova tumors treated with YCH1899, DMXAA, or their combination. Left, representative images of immunohistochemical staining; scale bar: 100 μm. Right, quantification of average staining intensity for CD45, CD4, and CD8. Data are presented as mean ± SEM; n ≥ 6 technical replicates per group; ***, *p* < 0.001 (one-way ANOVA)

Given that YCH1899 potentiated cGAMP-induced interferon signaling in vitro, we next evaluated whether combining YCH1899 with a STING agonist could enhance antitumor efficacy in vivo. We first used the syngeneic CT26 colon carcinoma model, a microsatellite-stable (MSS) tumor model with intrinsic immunogenicity but limited responsiveness to PD-1 blockade [37]. DMXAA, a murine-specific STING agonist widely used in preclinical studies [38], was used to activate STING signaling in vivo. CT26 cells were implanted subcutaneously into BALB/c mice, which were then randomized to receive vehicle, YCH1899, DMXAA, or the combination according to the treatment schedule shown in Fig. 5a.

The findings indicated that while DMXAA alone led to noticeable tumor growth inhibition, the combined treatment with YCH1899 and DMXAA resulted in enhanced tumor growth suppression. In contrast, YCH1899 monotherapy exhibited moderate antitumor efficacy (Fig. 5b and Fig. S5a). Combination efficacy was quantified using invivoSyn [39]. The highest single agent (HSA) model implemented in the CombPDX algorithm yielded an HSA combination index of 1.33 (*p* < 0.01), corresponding to a 74% reduction in relative tumor volume compared with DMXAA monotherapy. Additionally, the tumor weight was significantly lower in the combination group than in the vehicle or either monotherapy group (Fig. S5a). We next evaluated the combination in the independent MC38-Ova model. YCH1899 monotherapy significantly suppressed MC38-Ova tumor growth, and the addition of DMXAA further enhanced tumor growth inhibition compared with either agent alone (Fig. 5c and Fig. S5b). The HSA combination index reached 1.19 at the study endpoint (*p* < 0.05).

To investigate the immunological basis of the enhanced antitumor activity, we analyzed immune cell infiltration and activation within the tumor microenvironment. Flow cytometric analysis of CT26 tumors showed that combination treatment significantly increased the frequency and activation of tumor-infiltrating CD8⁺ T cells, accompanied by a modest increase in granzyme B expression in the CD4⁻CD8⁺ T-cell population (Fig. 5d). Similarly, combination therapy increased the infiltration of CD45⁺ leukocytes and CD4⁺ and CD8⁺ T cells in MC38-Ova tumors relative to either monotherapy (Fig. 5e). These findings demonstrate that YCH1899 cooperates with STING agonists to enhance antitumor immunity and tumor control in vivo.

## Discussion

Approved PARP inhibitors, such as olaparib and talazoparib, have been shown to induce DNA damage and prime interferon pathway activation in BRCA-deficient cells [5, 6], whereas only limited activation is observed in BRCA-proficient tumors. In this study, we demonstrate that the next-generation PARP inhibitor YCH1899 robustly activates the interferon pathway in vitro and suppresses the growth of multiple BRCA-proficient tumors in vivo. This antitumor activity is accompanied by increased infiltration of CD45^+^ leukocytes and CD8^+^ T cells. These findings extend the immunotherapeutic potential of PARP inhibitors beyond BRCA-deficient cancers.

While most PARP inhibitors effectively block poly(ADP-ribosyl)ation, they differ markedly in their capacity to trap PARP1 on DNA [40]. Talazoparib, recognized for potent PARP–DNA trapping activity, was demonstrated to activate innate immunity in BRCA-proficient tumor cells [9]. Compared with talazoparib, YCH1899 induces greater DNA damage, upregulates interferon pathway gene expression, and enhances phosphorylation of IRF3 and STAT1 (Fig. 1c, d, 2a). Our previous work revealed that YCH1899 exhibited a 100-fold greater trapping potency than olaparib [24], supporting the hypothesis that its enhanced DNA damage underlies superior antitumor efficacy in BRCA-proficient tumors. Notably, YCH1899 retains activity in PARP inhibitor-resistant tumor models, including those with BRCA1/2 restoration or 53BP1 loss [24]. This enhanced PARP-trapping capacity and ability to overcome resistance distinguish YCH1899 from classical PARP inhibitors such as olaparib, providing a mechanistic basis for its improved efficacy in BRCA-proficient tumors.

To elucidate regulators of PARP inhibitor-mediated antitumor immunity, we employed pooled in vivo loss-of-function CRISPR screens. Deficiencies in specific DNA damage repair genes, including *Rad51c*, *Pnkp*, and *Atm*, enhanced tumor sensitivity to YCH1899, whereas the deletion of interferon pathway genes, including *Irf7*, *Nod2* and *Irf3*, attenuated its antitumor effects (Fig. 3b). Notably, USP20 deletion significantly diminished tumor response to YCH1899 (Fig. 3g). USP20 is reported to be involved in STING deubiquitination, thereby promoting innate antiviral responses [34, 35]. However, our data indicate that changes in STING expression were not as significant as the attenuation of interferon gene expression or STAT1 phosphorylation (Fig. 3e). To explore the mechanisms underlying USP20 regulation of STING pathway activation, we performed proteomic analysis in USP20-deficient cells and identified immune-related proteins, including DPY30, PJA1, and SERPINE1, as potential USP20 substrates (Fig. S3c). These findings suggest that USP20 may modulate antitumor immunity through multiple downstream effectors, although the precise mechanisms require further investigation. Moreover, low *USP20* expression is associated with poor survival, suggesting its potential prognostic relevance. USP20 may also serve as a predictive biomarker of PARP inhibitor response, and maintaining or enhancing USP20 activity could improve responses to PARP inhibition.

To further potentiate YCH1899-mediated immune responses, we investigated its combination with STING agonists. YCH1899 significantly amplified STING-induced interferon signaling and inhibited tumor growth in CT26 and MC38-Ova syngeneic models. Consistently, this combination therapy increased tumor-infiltrating CD8^+^ T cells and enhanced granzyme B expression (Fig. 5d). Mechanistically, YCH1899 induced phosphorylation of IRF3 at Ser173, which may facilitate IRF3 dimerization and subsequent Ser396 phosphorylation, thereby amplifying STING pathway activation under combination treatments.

In BRCA-deficient tumors, an immunosuppressive microenvironment can limit the efficacy of PARP inhibitors [41]. Moreover, downregulation of interferon signaling has been observed in patients with acquired resistance to PARP inhibition (Fig. 4a). These findings suggest that the combination of YCH1899 and STING agonists has the potential to overcome immune evasion and enhance therapeutic responses in immunosuppressed or PARP inhibitor–resistant tumors. By enabling tumor-restricted immune activation while minimizing systemic toxicity through the use of low-dose STING agonists, the YCH1899–STING agonist combination represents a translationally relevant strategy to extend the therapeutic benefit of PARP inhibition to both BRCA-proficient tumors and immunosuppressed BRCA-deficient malignancies. Further validation in clinically relevant models, including patient-derived xenografts and organoid systems, will be required to substantiate the efficacy and translational potential of this approach.

## Conclusions

Overall, this study demonstrates the therapeutic potential of YCH1899 as a next-generation PARP inhibitor that elicits robust antitumor immune responses in BRCA-proficient tumors. Through enhanced PARP trapping and DNA damage induction, YCH1899 activates interferon signaling in a STING-dependent manner, leading to effective tumor suppression in vivo. Using in vivo CRISPR screens, we identified USP20 as a critical regulator of YCH1899-induced innate immune activation, suggesting potential biomarker utility. Moreover, YCH1899 promotes phosphorylation of IRF3 at Ser173, thereby potentiating STING agonist-induced interferon signaling and producing synergistic antitumor effects. Collectively, these findings support the use of YCH1899 either as a monotherapy or in combination with STING agonists to overcome immune suppression and extend the clinical benefit of PARP inhibition to BRCA-proficient and PARP inhibitor-resistant tumors.

## Materials and methods

### Compounds and antibodies

YCH1899 was synthesized by the Shanghai Institute of *Materia Medica*, Chinese Academy of Sciences [24]. Olaparib, niraparib, RBN-2397, and digitonin were obtained from MedChemExpress (MCE). Talazoparib was purchased from Selleck Chemicals. 2',3'-cGAMP was sourced from InvivoGen. DMXAA was acquired from Giant Pharmaceutical Technology and Selleck Chemicals. Unless otherwise specified, compounds were dissolved in dimethyl sulfoxide (DMSO), aliquoted, and stored at ˗40 °C.

The β-actin antibody (#60008) was obtained from Proteintech, the GAPDH antibody (#AF0006) from Beyotime, and the NF-κB p65 antibody (#sc-514451) from Santa Cruz Biotechnology. Antibodies against lamin B1 (#A11495) and cGAS (#A8335) were obtained from ABclonal. Antibodies obtained from Cell Signaling Technology included PARP (#9542), phospho-STAT1 (Tyr701) (#9167), STAT1 (#14994), TBK1 (#3504), phospho-TBK1 (Ser172) (#5483), STING (#13647), phospho-IRF3 (Ser396) (#4947), and IRF3 (#11904). Horseradish peroxidase (HRP)-conjugated goat anti-mouse IgG (#115-035-003) and goat anti-rabbit IgG (#111-035-003) were obtained from Jackson ImmunoResearch Laboratories.

For flow cytometry, the following fluorochrome-conjugated anti-mouse antibodies were used: FITC anti-CD45 (#553079, BD Biosciences), Pacific Blue anti-CD4 (#100531, BioLegend), BV605 anti-CD3 (#100237, BioLegend), PerCP anti-CD8a (#100731, BioLegend), PE anti-PD-1 (#135205, BioLegend), and PE-Cy7 anti-granzyme B (#372213, BioLegend). Purified rat anti-mouse CD16/CD32 (#553141, BD Biosciences) was used to block Fc receptors before staining.

For immunohistochemical staining, antibodies against mouse CD4 (#ab288724, Abcam), CD8 (#ab237709, Abcam), and CD45 (#ab10558, Abcam) were used.

### Cell lines

Human HT-29 and HeLa cells were obtained from the American Type Culture Collection (ATCC) and cultured in McCoy’s 5 A medium and minimum essential medium (MEM), respectively, each supplemented with 10% fetal bovine serum (FBS). 293T cells were acquired from Obio Technology and cultured in Dulbecco’s modified Eagle medium (DMEM) supplemented with 10% FBS. MC38-Ova cells, provided by Dr. Junyi Chen, were cultured in DMEM supplemented with 10% FBS. CT26 and MC38 cells were acquired from EK-Bioscience and cultured in RPMI-1640 and DMEM, respectively, each supplemented with 10% FBS. 4T1 and LL/2 cells were obtained from ATCC and MeilunBio, respectively, and cultured in RPMI-1640 and DMEM, respectively. All cell lines were authenticated by short tandem repeat (STR) analysis performed by Genesky or Genetic Testing Biotechnology Corporation or by SNP-NGS analysis performed by Crown Bioscience.

### Western blotting

Cells were seeded in 12-well plates and treated with YCH1899 or the indicated compounds. Cells were then lysed in protein loading buffer (50 mM Tris–HCl, pH 6.8, 2% SDS, 0.05% bromophenol blue, 10% glycerol, and 100 mM dithiothreitol [DTT]) supplemented with protease and phosphatase inhibitors (MCE). Lysates were boiled for 10 min and clarified by centrifugation. Equal volumes of the resulting supernatants were loaded into each lane. Proteins were separated by sodium dodecyl sulfate–polyacrylamide gel electrophoresis (SDS-PAGE) and transferred to 0.45-µm nitrocellulose membranes using the Trans-Blot Turbo system (Bio-Rad). Membranes were blocked with 5% nonfat milk for 1 h and incubated with primary antibodies at 4 °C overnight. After incubation with HRP-conjugated secondary antibodies at room temperature for 1 h, protein bands were visualized using the ChemiDoc imaging system (Bio-Rad).

### RNA sequencing (RNA-seq)

Total RNA was extracted from parental and PARP1-knockout HT-29 cells using a HiPure Total RNA Plus Kit (#R4111, Magen Biotechnology). RNA-seq library preparation and sequencing were performed by BGI Tech (Shenzhen, China). Libraries were sequenced on a BGI platform using a 50-bp single-end (SE50) strategy.

RNA-seq data were analyzed by BGI Tech or in-house. For the in-house analysis, raw-read quality was assessed using FastQC and MultiQC. Adapter sequences and low-quality bases were removed using Cutadapt, after which reads were aligned to the human reference genome (GRCh38) using HISAT2. Gene-level read counts were generated using featureCounts with GENCODE gene annotations. Lowly expressed genes were filtered out, and normalization factors were calculated using the trimmed mean of M-values (TMM) method. Differential expression analysis was performed using the edgeR package in R [42]. Where indicated, Reactome pathway enrichment analysis was performed using differential gene lists provided by BGI Tech. Volcano plots were generated from the in-house differential expression results using ggplot2.

### In vivo antitumor activity

C57BL/6J, BALB/c, and BALB/c nude mice were obtained from HFK Bioscience (Beijing, China) and maintained under specific pathogen-free conditions. MC38-Ova cells (2 × 10⁶), CT26 cells (1 × 10^5^), MC38 cells (1 × 10^5^), 4T1 cells (1 × 10^5^), or LL/2 cells (1.5 × 10^5^) were injected subcutaneously into the axillary region. Once tumors became palpable, mice were randomly assigned to groups and received YCH1899 by oral gavage, DMXAA by intratumoral injection, or the combination according to the indicated treatment schedules. YCH1899 formulations were prepared as previously described [24], and DMXAA was dissolved in 5% NaHCO_3_. Tumor volume and body weight were measured twice weekly. Relative tumor volume (RTV) was calculated by normalizing the tumor volume at each time point to that at treatment initiation (day 0). Tumor growth inhibition was expressed as the ratio of the RTV in the treatment group to that in the vehicle-control group (T/C, %). For prophylactic treatment models, endpoint T/C (%) was calculated as treatment-to-control tumor volume ratio. All animal experiments were conducted in accordance with the guidelines of the Institutional Animal Care and Use Committee (IACUC) of the Shanghai Institute of *Materia Medica*. Animals were euthanized when tumor volume reached 2,000 mm^3^.

### Analysis of tumor-infiltrating lymphocytes by flow cytometry

At the endpoint of the CT26 model study, tumors were harvested, mechanically sectioned, and transferred to gentleMACS C tubes. The tumor fragments were subsequently dissociated into single-cell suspensions using the Tumor Dissociation Kit (#130-096-730, Miltenyi Biotec) with the enzyme buffer on a gentleMACS Octo Dissociator (Miltenyi Biotec). The resulting suspensions were filtered through a 70-µm cell strainer, and any remaining red blood cells were lysed using BD Pharm Lyse Lysing Buffer (#555899, BD Biosciences). Non-viable cells were excluded by employing the FVS780 viability dye (#565388, BD Biosciences), followed by an Fc Receptor block and staining with surface antibodies at 4 °C for 30 min. After washing, the cells were fixed and permeabilized with Cytofix/Cytoperm Fixation/Permeabilization Kit (#554714, BD Biosciences) in accordance with the manufacturer’s protocol. Intracellular staining was then performed at 4 °C for 30 min. The cells were analyzed using an LSRFortessa flow cytometer. Compensation was performed using single-stained controls. Data were processed using FlowJo v10 software, incorporating the FlowAI plugin to exclude aberrant events [43].

### 2',3'-cGAMP stimulation

Stimulation of cells with 2',3'-cGAMP was conducted in accordance with previously established protocols [44]. Cells were treated with the specified compounds for 48 h. Following a wash with PBS, 0.02 µM of 2',3'-cGAMP was introduced into the cells in Buffer A (50 mM HEPES–KOH, pH 7.2, 100 mM KCl, 3 mM MgCl₂, 0.1 mM DTT, 85 mM sucrose, 0.2% BSA, and 1 mM ATP) containing digitonin (10 µg/mL) for 15 min. Subsequently, the cells were rinsed with PBS, cultured in fresh medium, and lysed at predetermined time points for immunoblotting or real-time qPCR.

### Statistical analyses

Statistical analyses were performed using GraphPad Prism 9. For comparisons involving two or three groups, either an unpaired two-tailed Student’s t-test or ANOVA was employed. The synergistic effect of the in vivo combination treatment was evaluated using the day-specific combination index (CI) calculated by the CombPDX method in the R package invivoSyn under the highest single agent (HSA) reference model; the analysis yielded the CI and corresponding p-value [39]. Statistical significance was indicated as follows: ns, not significant; *p < 0.05; **p < 0.01; ***p < 0.001. Unless otherwise specified, the representative results depicted in each figure were obtained from two or three independent experiments. Data are presented as the mean ± SEM unless otherwise indicated.

## Supplementary Information

Supplementary Material 1.

Supplementary Material 2.

Supplementary Material 3.

Supplementary Material 4.

Supplementary Material 5.

## Data Availability

The raw RNA-seq data generated in this study were deposited in the Genome Sequence Archive of the National Genomics Data Center under accession number HRA019970 (GSA-Human) and are publicly accessible at https://ngdc.cncb.ac.cn/gsa-human. Additional datasets generated or analyzed during this study are included in the Supplementary Information or are available from the corresponding author on reasonable request.

## Abbreviations

cGAMP: Cyclic GMP-AMP
cGAS: Cyclic GMP-AMP synthase
CI: Combination index
DMXAA: 5,6-Dimethylxanthenone-4-acetic acid
HSA: Highest single agent
IFNAR: Type I interferon receptor
ISG: Interferon-stimulated gene
PARP: Poly(ADP-ribose) polymerase
RNA-seq: RNA sequencing
RTV: Relative tumor volume
STING: Stimulator of interferon genes
T/C: Treatment-to-control ratio
