# Supplementary material for: PARP inhibitor YCH1899 activates type I interferon signaling and synergizes with STING agonists in BRCA-proficient tumors
Source: Mol Biomed. 2026 Aug 26;7:151. doi: 10.1186/s43556-026-00553-x (PMC13518744; doi:10.1186/s43556-026-00553-x)
Supplement: Supplementary file 1 [file 43556_2026_553_MOESM1_ESM.docx]

PARP Inhibitor YCH1899 Activates Type I Interferon Signaling and Synergizes with STING Agonists in BRCA-Proficient Tumors

Li Jia^1,2#^, Ming-Hao Cao^1,2#^, Yu-Ting Sun^2,3^, Xu-Bin Bao^1,2^, Wen-Ting Zhu^1,2^, Yong-Jun Cheng^1,2^, Li-Min Wang^1,2^, Hui Yang^1,2^, Shan-Shan Song^1,2^, Chang-Qing Tian^1,2^, Ze-Hong Miao^1,2^, Chun-Hao Yang^2,3^, Jin-Xue He^1,2^

**Authors’ Affiliations:**

^1^State Key Laboratory of Drug Research, Cancer Research Center, Shanghai Institute of *Materia Medica*, Chinese Academy of Sciences, Shanghai 201203, China;

^2^University of Chinese Academy of Sciences, No.19A Yuquan Road, Beijing 100049, China;

^3^State Key Laboratory of Drug Research, Department of Medicinal Chemistry, Shanghai Institute of *Materia Medica*, Chinese Academy of Sciences, Shanghai, 201203, China；

(^#^ These authors contributed equally to this work)

**Corresponding Authors:**

Dr. Jin-Xue He, E-mail: [jinxue_he@simm.ac.cn](mailto:jinxue_he@simm.ac.cn).

Supplementary methods

Real-time quantitative PCR

Cells cultured in 12-well plates were treated with YCH1899 or other specified compounds. Total RNA was extracted using the SteadyPure Universal RNA Extraction Kit (#AG21017, Accurate Biology). Complementary DNA (cDNA) was synthesized from 1 µg of total RNA using the HiScript III RT SuperMix Reverse Transcription Kit (#R323-01, Vazyme). Real-time quantitative PCR (qPCR) was performed using SYBR Green Premix Pro Taq (#AG11701, Accurate Biology) and gene-specific primers on an Applied Biosystems 7500 Real-Time PCR System or a Bio-Rad CFX96 Real-Time PCR Detection System. The primer sequences used in this study are listed below:

For human cells:

*IFNB1*-F: 5’-ATGACCAACAAGTGTCTCCTCC-3’

*IFNB1*-R: 5’-GGAATCCAAGCAAGTTGTAGCTC-3’

*CXCL10*-F: 5’-GAACTGTACGCTGTACCTGCA-3’

*CXCL10*-R: 5’-TTGATGGCCTTCGATTCTGGA-3’

*MX1*-F: 5’-GGCTGTTTACCAGACTCCGACA-3’

*MX1*-R: 5’-CACAAAGCCTGGCAGCTCTCTA-3’

*MX2*-F: 5’-AAAAGCAGCCCTGTGAGGCATG-3’

*MX2*-R: 5’-GTGATCTCCAGGCTGATGAGCT-3’

*CCL5*-F: 5’-GCTGTCATCCTCATTGCTACTG-3’

*CCL5*-R: 5’-TGGTGTAGAAATACTCCTTGATGTG-3’

*GAPDH*-F: 5’-GTCTCCTCTGACTTCAACAGCG-3’

*GAPDH*-R: 5’-ACCACCCTGTTGCTGTAGCCAA-3’

For murine cells:

*Ifnb1*-F: 5’-CAGCTCCAAGAAAGGACGAAC-3’

*Ifnb1*-R: 5’-GGCAGTGTAACTCTTCTGCAT-3’

*Cxcl10*-F: 5’-CCAAGTGCTGCCGTCATTTTC-3’

*Cxcl10*-R: 5’-GGCTCGCAGGGATGATTTCAA-3’

*Gapdh*-F: 5’-GGTCCCAGCTTAGGTTCATCA-3’

*Gapdh*-R: 5’-CCCAATACGGCCAAATCCGT-3’

Generation of gene knockout cells using CRISPR/Cas9

The CRISPR/Cas9 system was employed to generate the gene-knockout cell lines used in this study. Briefly, HEK293T cells were seeded in 6-well plates and grown to approximately 80% confluence before transfection. Lentiviral particles were produced by co-transfecting pLentiCRISPRv2-sgRNA, psPAX2, and pMD2.G at a plasmid mass ratio of 4:2:1 (total DNA, 2.5 µg per well) using PEI (10 µg) according to the manufacturer’s instructions. The medium was replaced 6 h after transfection. Viral supernatants were collected 48 h after transfection, centrifuged at 1,000 × g for 5 min to remove cell debris, and filtered through a 0.45-µm filter. Target cells were seeded in 6-well plates and grown to approximately 60% confluence before infection. The cells were then infected with 600 µL of lentiviral supernatant supplemented with polybrene (8 µg/mL) for 12 h. At 48 h post-infection, the medium was replaced with fresh medium containing puromycin at 1.5 or 2 µg/mL, and selection was continued for 7 days.

To derive single-cell knockout clones, puromycin-selected cells were seeded at a density of 0.8 cells per well in 96-well plates and expanded. Gene disruption was confirmed by Western blotting and Sanger sequencing. For Sanger sequencing, genomic DNA was extracted, and the target locus was amplified by PCR. Sanger sequencing results were analyzed using the Inference of CRISPR Edits (ICE) tool. The construction of PARP1/PARP7 double-knockout cells has been described previously [1]. The sequences of the sgRNAs utilized in this study are provided below:

Human *CGAS*: 5’-CTTTCCGTGCCAAGGCTGCA-3’

Human *STING1*: 5’-GGCTGTCACTCACAGGTACC-3’

Human *IRF3*: 5’-CAGAACCAGAGGGCATAGCG-3’

Human *PARP1*-1: 5’-CGATGCCTATTACTGCACTG-3’

Human *PARP1*-2: 5’-ACCCTGACGTTGAGGTGGAT-3’

Human *PARP7:* 5’-CACTGAAGCTCCAGAACGAG-3’

Mouse Sting1: 5’-GCTGGATGCAGGTTGGAGTA-3’

Mouse *Irf3*: 5’-AATCCCTGATATTTCAGGCC-3’

Mouse *Usp20*: 5’-TTGGTCAACCCGATGTTCCG-3’.

PicoGreen staining assay

Micronucleus formation was evaluated using a PicoGreen staining assay. Cells were exposed to the specified compounds for 48 h. PicoGreen dye (P7581, Invitrogen) was then added to the medium at a 1:1,000 dilution, and the cells were incubated for an additional 1 h before fixation. Cells were fixed with 4% paraformaldehyde at room temperature for 15 min and permeabilized with 0.5% Triton X-100 for 20 min. Following fixation and permeabilization, nuclei were stained with DAPI. Images were captured using a confocal laser-scanning microscope (LSM 920, Zeiss).

Immunohistochemical staining

Tumors were fixed in 4% paraformaldehyde, and subsequent paraffin embedding, sectioning, and staining were performed by RIBIOLOGY (Shanghai, China) or ZuoChengBio (Shanghai, China). Slides were imaged using either an FF-PRO-120 system or a Leica DM6 B microscope. Images were then analyzed using Fiji software. Target staining was quantified as the mean optical density, defined as the integrated optical density (IOD) divided by the area of the positively stained region.

Immunoprecipitation

Cells were lysed in immunoprecipitation lysis buffer (25 mM Tris-HCl, pH 7.4; 150 mM NaCl; 1 mM EDTA; 1% NP-40; and 5% glycerol) supplemented with protease and phosphatase inhibitors. Lysates were sonicated for 12 s, incubated on ice for 20 min, and then centrifuged to clarify the supernatants. The supernatants were pre-cleared with magnetic beads (#70024, CST) and incubated with the indicated antibody at 4°C overnight. The lysates were then incubated with pre-washed magnetic beads at 4°C for 2 h. Beads were collected using a magnetic rack and washed with lysis buffer. The bead-bound proteins were either subjected to mass spectrometry analysis or mixed with 5× protein loading buffer, boiled, and analyzed by SDS-PAGE.

Mass spectrometry analysis

Mass spectrometry analysis was performed by the Public Technology Service Center, Shanghai Institute of *Materia Medica*, Chinese Academy of Sciences. Sample preparation and data acquisition were performed as previously described [2].

Briefly, for proteomic analysis of USP20 deficiency, parental and USP20-knockout MC38-Ova cells were lysed in SDT lysis buffer (4% SDS, 0.1 M Tris-HCl, 0.1 M DTT, pH 7.6). Protein concentrations were determined using a tryptophan fluorescence-based assay, and 50 µg of protein per sample was used for proteomic analysis. Peptide concentrations were quantified by NanoDrop before injection. For DIA acquisition, 1 µg of peptide sample was loaded and analyzed using a 90-min gradient on a Q Exactive HFX mass spectrometer (Thermo Fisher Scientific). Raw files were searched using DIA-NN v1.8.1. Identifications were filtered at a protein false discovery rate (FDR) < 0.01 to obtain high-confidence qualitative and quantitative results. Differentially expressed proteins were identified using the limma package in R.

For identification of IRF3 phosphorylation sites, YCH1899-treated HT-29 cells were lysed and subjected to immunoprecipitation using an anti-IRF3 antibody as described above. Bead-bound proteins were analyzed by LC–MS/MS on a Q Exactive HF mass spectrometer (Thermo Fisher Scientific). Sample preparation was performed as previously described [3], and raw files were searched using MaxQuant v2.4.10.0 with FDR < 0.01.

Drug resistance signature analysis

To analyze gene signatures associated with clinical resistance to PARP inhibitors, we used the CTR-DB database [4]. Samples from GSE194040 (ID: CTR_Microarray_161-I) were included, comprising 71 breast cancer samples (27 "non-responders" and 44 "responders") treated with carboplatin, paclitaxel, and veliparib. Differential gene expression analysis between non-responders and responders was performed as previously described [4].

Genome‐wide CRISPR-Cas9 knockout library screening

The CRISPR screening study was executed following a previously established method [5]. For the first-round genome-wide screen, the mouse GeCKO v2 lentiviral library was obtained from OBIO Technology. MC38-Ova cells were transduced with the viral library at a multiplicity of infection (MOI) of 20, resulting in approximately 30% cell survival after selection with puromycin at 2.5 µg/mL. The surviving cells were cultured in the presence of puromycin for 7 days to facilitate gene editing. Subsequently, the cells were transplanted subcutaneously into C57BL/6J mice at 2 × 10⁶ cells per mouse, with six mice per group, ensuring approximately 178-fold coverage of the library.

At the treatment endpoint, tumors were harvested, and genomic DNA was extracted following previously described protocols [6]. For the preparation of the sequencing library, two rounds of PCR were conducted. In the first round, the region containing sgRNA sequences was amplified from the genomic DNA using primers (forward: 5’-AATGGACTATCATATGCTTACCGTAACTTGAAAGTATTTCG-3’; reverse: 5’-TAGGCACCGGATCAATTGCCGAC-3’) and KAPA HiFi polymerase (#KK2502, Roche). Ten reactions were performed in accordance with the manufacturer’s instructions, with up to 20 cycles of amplification to produce discernible PCR product bands. To ensure over 150-fold library coverage, 120 µg of genomic DNA was utilized (12 µg per reaction). First-round PCR products were pooled and purified using VAHTS DNA Clean Beads (#N411, Vazyme). In the second round, sequencing adapters were added to 10 ng of purified PCR products through 8 cycles of amplification. Subsequently, residual primer oligonucleotides were digested using Exonuclease I (#EN0581, Thermo Fisher Scientific). The resulting second-round PCR products underwent size selection with VAHTS DNA Clean Beads, and their concentrations were quantified using a Qubit fluorometer. Sequencing was performed by Novogene using the Illumina NovaSeq system. The sequencing data were analyzed using MAGeCK and MAGeCK-VISPR software [7, 8].

For the second-round screening, a custom library was designed using the CRISPOR website [9], targeting 578 genes with approximately 10 sgRNA sequences per gene. The library consisted of 5,766 sequences, including 300 non-targeting sgRNAs. The oligonucleotide pool was synthesized by GenScript and subsequently amplified using specific primers (forward: 5’-TATCTTGTGGAAAGGACGAAACACC-3’; reverse: 5’-GCCTTATTTTAACTTGCTATTTCTAGCTCTAAAAC-3’). The PCR product was then cloned into the digested lentiCRISPRv2 vector using the Hieff Clone Plus One Step Cloning Kit (#10911ES20, Yeasen). The reaction products were purified with isopropanol, dissolved in water, and electroporated into Endura electrocompetent cells (#60240, Lucigen) following the manufacturer’s instructions. The transformed cells were plated on LB Lennox agar and incubated overnight for growth. Colonies were pooled, and plasmids were extracted. The custom plasmid library underwent quality control analysis as previously described [5]. After quality verification, the plasmid library was transfected into HEK293T cells to generate the viral library following the protocol outlined above. Subsequent procedures, including viral library infection, antibiotic selection, and transplantation, were conducted in accordance with the first-round screening.

Genome editing for single-base substitution

To construct IRF3 S173A knock-in cells, we performed genome editing using a CRISPR ribonucleoprotein (RNP) complex. The IRF3-targeting sgRNA was synthesized by GenScript (Nanjing, China) (5’–3’: GTCCAAGCTGGGGCTCCGCA). A single-stranded oligodeoxynucleotide (ssODN) donor template was synthesized by Sangon Biotech (Shanghai, China) (5’–3’: AAGCCTGGCT GTAGCCCCTG AGCCCTGCCC TCAGCCCCTG CGGGCCCCCA GCTTGGACAA TCCCACTCCC TTCCCAAACC TGGGGCCC). For RNP transfection, 7.5 pmol Cas9 protein (#A36497, Thermo Fisher Scientific) was mixed with 22.5 pmol sgRNA in Buffer R and incubated at room temperature for 10 min. Then, 1 µg of ssODN was added to bring the total volume to 7 µL, and the mixture was incubated for 2 min. Subsequently, 5 µL of an HT-29 cell suspension (5 × 10^6^ cells/mL) was added to the complex and electroporated using the Neon Transfection System (Thermo Fisher Scientific) under the following conditions: 1,650 V, 10 ms, and 2 pulses. Cells were cultured for 24 h and seeded into 96-well plates at a density of 0.8 cells per well. Single-cell clones were screened by Western blotting using an anti-IRF3 antibody; clones showing a reduced upper IRF3 band were further confirmed by Sanger sequencing. The editing outcomes were analyzed using the ICE tool.

Cell viability assays

Parental and USP20-knockout MC38-Ova cells were seeded into 96-well plates. After 24 h, cells were treated with the specified concentrations of YCH1899 for 6 days, followed by sulforhodamine B (SRB) assays, as previously described [10]. Optical density (OD) values were measured using a SpectraMax 190 Microplate Reader (Molecular Devices) and analyzed by non-linear regression using a four-parameter model.

Preparation of cytoplasmic and nuclear extracts

Cytoplasmic and nuclear extracts were prepared using the Nuclear and Cytoplasmic Protein Extraction Kit (#P0027, Beyotime) according to the manufacturer’s instructions. The resulting extracts were mixed with 5× loading buffer, boiled, and analyzed by Western blotting.

Native PAGE analysis

Cells were exposed to the indicated compounds for 48 h. After washing, cells were lysed in buffer containing 25 mM Tris-HCl (pH 7.4), 5% glycerol, 150 mM NaCl, 1% NP-40, and 1 mM EDTA. The lysates were mixed with 5× native sample loading buffer (#C506032-0005, Sangon Biotech) and analyzed by native PAGE, as previously described [11].

**References**

1.	Wang LM, Wang P, Chen XM, Yang H, Song SS, Song Z, et al. Thioparib inhibits homologous recombination repair, activates the type I IFN response, and overcomes olaparib resistance. EMBO Mol Med. 2023;15(3):e16235. https://doi.org/10.15252/emmm.202216235.

2.	Liu Q, Zhang J, Guo C, Wang M, Wang C, Yan Y, et al. Proteogenomic characterization of small cell lung cancer identifies biological insights and subtype-specific therapeutic strategies. Cell. 2024;187(1):184-203.e28. https://doi.org/10.1016/j.cell.2023.12.004.

3.	Johnston HE, Yadav K, Kirkpatrick JM, Biggs GS, Oxley D, Kramer HB, et al. Solvent Precipitation SP3 (SP4) Enhances Recovery for Proteomics Sample Preparation without Magnetic Beads. Anal Chem. 2022;94(29):10320-28. https://doi.org/10.1021/acs.analchem.1c04200.

4.	Jiang J, Ma Y, Yang L, Ma S, Yu Z, Ren X, et al. CTR-DB 2.0: an updated cancer clinical transcriptome resource, expanding primary drug resistance and newly adding acquired resistance datasets and enhancing the discovery and validation of predictive biomarkers. Nucleic Acids Res. 2024;53(D1):D1335-D47. https://doi.org/10.1093/nar/gkae993.

5.	Joung J, Konermann S, Gootenberg JS, Abudayyeh OO, Platt RJ, Brigham MD, et al. Genome-scale CRISPR-Cas9 knockout and transcriptional activation screening. Nat Protoc. 2017;12(4):828-63. https://doi.org/10.1038/nprot.2017.016.

6.	Wang X, Tokheim C, Gu SS, Wang B, Tang Q, Li Y, et al. In vivo CRISPR screens identify the E3 ligase Cop1 as a modulator of macrophage infiltration and cancer immunotherapy target. Cell. 2021;184(21):5357-74.e22. https://doi.org/10.1016/j.cell.2021.09.006.

7.	Li W, Xu H, Xiao TF, Cong L, Love MI, Zhang F, et al. MAGeCK enables robust identification of essential genes from genome-scale CRISPR/Cas9 knockout screens. Genome Biol. 2014;15(12):554. https://doi.org/10.1186/s13059-014-0554-4.

8.	Li W, Köster J, Xu H, Chen CH, Xiao TF, Liu JS, et al. Quality control, modeling, and visualization of CRISPR screens with MAGeCK-VISPR. Genome Biol. 2015;16:281. https://doi.org/10.1186/s13059-015-0843-6.

9.	Concordet J-P, Haeussler M. CRISPOR: intuitive guide selection for CRISPR/Cas9 genome editing experiments and screens. Nucleic Acids Res. 2018;46(W1):W242-W45. https://doi.org/10.1093/nar/gky354.

10.	Chen HD, Chen CH, Wang YT, Guo N, Tian YN, Huan XJ, et al. Increased PARP1-DNA binding due to autoPARylation inhibition of PARP1 on DNA rather than PARP1-DNA trapping is correlated with PARP1 inhibitor's cytotoxicity. Int J Cancer. 2019;145(3):714-27. https://doi.org/10.1002/ijc.32131.

11.	Yan Z, Liu H, Gao C. Native-PAGE analysis of protein aggregation upon viral infection in mouse macrophages. STAR Protoc. 2022;3(1):101080. https://doi.org/10.1016/j.xpro.2021.101080.

Supplementary figures

**Fig. S1 PARP1 deficiency activates interferon signaling, and YCH1899 elicits interferon responses and antitumor activity in BRCA-proficient models.** **a** Volcano plot showing differentially expressed genes in PARP1-KO HT-29 cells compared with parental HT-29 cells. Red dots represent significantly upregulated genes (*p* < 0.05, log2 fold change > 1) in PARP1-KO cells, and blue dots represent significantly downregulated genes (*p* < 0.05, log2 fold change < −1). **b** PARP1 knockout increases STAT1 phosphorylation in HT-29 cells. **c** YCH1899 and talazoparib (TP) upregulated *CXCL10* and *Cxcl10* mRNA levels in HT-29 and MC38-Ova cells, respectively. Cells were treated with the indicated concentrations for 48 h. Data are presented as mean ± SEM. *, *p* < 0.05; **, *p* < 0.01 (n = 3, one-way ANOVA); n.s., not significant. **d** YCH1899 (1 μM) and talazoparib (10 μM) showed comparable antiproliferative effects. HT-29 and MC38-Ova cells were treated for 48 h, and cell viability was measured using CCK-8 assays. Data are presented as mean ± SEM (n=3). **e** Tumor volumes of MC38, 4T1, and LL/2 tumors in C57BL/6J or BALB/c mice. Mice were treated by oral administration of YCH1899 at 20 mg/kg for 14 days (MC38, n = 6 mice per group), 15 mg/kg for 10 days (4T1, n = 10 mice per group), or 15 mg/kg for 13 days (LL/2, n = 8 mice per group). Data are presented as mean ± SEM. *, *p* < 0.05; and ***, *p* < 0.001 (two-way ANOVA); n.s., not significant.

**Fig. S2 Depletion of STING or IRF3 abolishes YCH1899-induced interferon pathway activation in BRCA-proficient cells. a** Knockout of STING or IRF3 impaired YCH1899-induced *CXCL10* upregulation in HT-29 cells. Cells were treated with 1 μM YCH1899 or 10 μM talazoparib (TP) for 48 h. Data are presented as mean ± SEM. **, *p* < 0.01 (n = 3, one-way ANOVA). **b** Depletion of STING or IRF3 abolished YCH1899-induced activation of the interferon pathway in MC38-Ova cells. Cells were treated with 1 μM YCH1899 for 48 h. **c** Depletion of STING or IRF3 abolished YCH1899-induced upregulation of *Ifnb1* and *Cxcl10* mRNA levels in MC38-Ova cells. Cells were treated with 1 μM YCH1899 for 48 h. Data are presented as mean ± SEM. **, *p* < 0.01 (n = 3, one-way ANOVA). **d** PARP1, but not PARP7, was essential for YCH1899-mediated activation of the interferon pathway. Cells were treated with 1 μM YCH1899, 10 μM talazoparib (TP), or 1 μM RBN-2397 for 48 h. Data are presented as mean ± SEM; n = 2 or 3.

**Fig. S3 Gene ranking of MAGeCK analysis results in YCH1899-treated mice, validation and analysis of USP20-knockout MC38-Ova cells. a** Gene ranking and log2 fold change in YCH1899-treated mice compared with vehicle-treated controls from whole-genome CRISPR screens analyzed by MAGeCK. Left, results from C57BL/6J mice, with genes highlighted in the RRA plot (Fig. 3**b**); right, results from BALB/c nude mice, with top enriched and depleted genes listed. **b** Sanger sequencing validation of USP20 knockout in MC38-Ova cells. Top, genomic DNA sequence near the sgRNA-targeted region (gray rectangle); bottom, Sanger sequencing chromatogram. **c** Proteomic analysis of differentially expressed proteins in USP20-knockout cells compared with parental MC38-Ova cells. Red dots represent significantly upregulated proteins (*p* < 0.05, fold change > 1.5) in USP20-knockout cells, and blue dots represent significantly downregulated proteins (*p* < 0.05, log2 fold change < −1.5) (n = 2 biological replicates).

**Fig. S4** **Identification of the contribution of IRF3 Ser173 phosphorylation to the combined effects of YCH1899 and cGAMP.** **a** Proteomic analysis of immunoprecipitated IRF3 from YCH1899-treated HT-29 cells identified two potential adjacent phosphorylated serine sites (Ser173 and Ser175). **b** The IRF3 S173A mutation diminished the upper phosphorylated band. 293T and HeLa cells were transfected with myc-IRF3, myc-IRF3 S173A, or myc-IRF3 S175A vectors for 48 h. **c** Sanger sequencing validation of the genomic IRF3 S173A mutation in HT-29 knock-in cells. Top, genomic DNA sequence near the sgRNA-targeted region in parental cells; bottom, sequences of the two alleles in knock-in cells. One contained a deletion mutation, and the other carried the intended alanine substitution at IRF3 Ser173.

**Fig. S5 *In vivo* impact of YCH1899 and DMXAA administration on tumor progression.** The antitumor efficacy of combined YCH1899 and DMXAA treatment was evaluated in syngeneic CT26 tumors in BALB/c mice (**a**) and MC38-Ova tumors in C57BL/6J mice (**b**). Upper panels, representative images of tumors after 14 days (**a**) or 21 days (**b**) of the indicated treatment. Lower panels, corresponding tumor weights for each treatment group. Data are presented as mean ± SEM; n = 6 mice per group; **, *p* < 0.01; ***, *p* < 0.001 (one-way ANOVA); n.s., not significant.
